# Transcriptomics age acceleration in prolonged treated HIV infection

**DOI:** 10.1111/acel.13951

**Published:** 2023-08-07

**Authors:** Flora Mikaeloff, Marco Gelpi, Alejandra Escos, Andreas D. Knudsen, Julie Høgh, Thomas Benfield, João Pedro de Magalhães, Susanne D. Nielsen, Ujjwal Neogi

**Affiliations:** ^1^ The Systems Virology Lab, Division of Clinical Microbiology, Department of Laboratory Medicine Karolinska Institutet Stockholm Sweden; ^2^ Copenhagen University Hospital Rigshospitalet Copenhagen Denmark; ^3^ Department of Infectious Diseases Copenhagen University Hospital Hvidovre Denmark; ^4^ Institute of Inflammation and Ageing University of Birmingham, Queen Elizabeth Hospital, Mindelsohn Way Birmingham UK

**Keywords:** biological aging, transcriptomics aging clock, treated HIV infection

## Abstract

Biological aging in people with HIV (PWH) with prolonged successful antiretroviral therapy (ART) is convoluted and poorly defined. Here, we aimed to investigate the transcriptomics age estimator (TAE) in a cohort of 178 PWH on prolonged successful ART with immune reconstitution and viral suppression from the Copenhagen Comorbidity (COCOMO) cohort. We also used 143 clinical, demographical, and lifestyle factors to identify the confounders potentially responsible or associated with age acceleration. Among the PWH, 43% had an accelerated aging process (AAP), and 21% had decelerated aging process (DAP). DAP is linked with older age, European ancestry, and higher use of tenofovir disoproxil/alafenamide fumarate. A directionally class‐based gene set enrichment analysis identified the upregulation of inflammatory pathways (e.g., cytokine and Retinoic acid‐inducible gene I (RIG‐I)‐like receptor signaling pathways) and immune response like T‐cell receptor signaling, antigen processing, and presentation in AAP and the downregulation of metabolic processes like oxidative phosphorylation, pyruvate metabolism.

## Graphical abstract

The study on people with HIV undergoing prolonged successful ART found that 43% experienced accelerated biological aging, while 21% had decelerated biological aging measured using the transcriptomics aging clock. Factors associated with decelerated aging included older age, European ancestry, and higher use of antiretroviral tenofovir (TDF/TAF). Gene analysis revealed the upregulation of inflammatory pathways and downregulation of metabolic processes in accelerated aging.

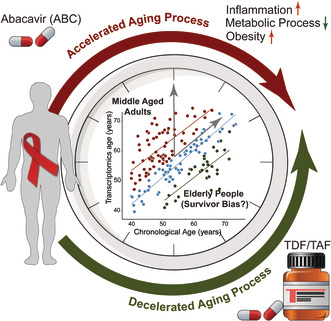

**Abbreviations**
AAP: accelerated aging process
ABC: abacavir
AGEs: advanced glycation end‐products
ART: antiretroviral therapy
AZT: zidovudine
BMI: body mass index
COCOMO: Copenhagen comorbidity cohort
d4T: stavudine
DAP: decelerated aging process
DDI: didanosine
DGE: differential gene expression
FN‐1: Fibronectin
GSE: gene set enrichment
OXPHOS: oxidative phosphorylation
PLCB2: phospholipase C beta 2
PWH: people living with HIV
PWoH: people without HIV
RAGE: receptor for advanced glycation end ‐products
RAP: regular aging process
RIG‐1: retinoic acid‐inducible gene I
SAT: subcutaneous adipose tissue
TAE: transcriptomics age estimator
TAF: tenofovir alafenamide
TDF: tenofovir disoproxil fumarate
VAT: visceral adipose tissue

The aging process in people living with HIV (PWH) on prolonged antiretroviral therapy (ART) is convoluted with evidence of premature, accentuated, and accelerated aging (De Francesco et al., 2019; Gooden et al., 2022). The biological age defined by epigenetic age was higher in PWH without therapy than in people without HIV (PWoH) (Esteban‐Cantos et al., 2021). Moreover, there was an early and considerable influence of HIV infection on the epigenetic aging process (Breen et al., 2022) that was partially reversed after ART initiation but remained significantly higher in PWH than in PWoH (Esteban‐Cantos et al., 2021). Though epigenetic clocks based on DNA methylation data have been widely used, they provide little information about the molecular and biological processes of the aging phenotype (Holzscheck et al., 2021). Theoretically, a molecular clock based on gene expression data can provide insights into the biological and cellular aging processes highly influenced by the environment, lifestyle, and medication. All the earlier studies (Breen et al., 2022; Esteban‐Cantos et al., 2021) on the biological age estimator in PWH were restricted in the number of clinical parameters with a shorter duration of treatment and limited by lack of lifestyle information. The impact of persistent HIV and long‐term ART on the biological age and its relationship to comorbidities remain undetermined. No transcriptomics data‐based age prediction has been thus far reported in PWH.

Here, we aimed to investigate the transcriptomics age estimator (TAE) in a cohort of 178 PWH who were on prolonged successful ART with immune reconstitution (increase in CD4) and viral suppression from a sub‐cohort of the Copenhagen Comorbidity (COCOMO) cohort. The sub‐cohort is representative of the main cohort with respect to sex, ethnicity, and HIV‐related parameters (Table S1). We calculated the transcriptomics age acceleration (delta age) and identified potential biological mechanisms behind the biological aging dysregulation in PWH. We also used 143 clinical, demographical, and lifestyle factors, including diet and their interactions, to understand potential factors responsible for the biological age acceleration.

We investigated four different TAE, BitAge (Meyer & Schumacher, 2021), BURNS (Shokhirev & Johnson, 2021), and RNAAgeCalc, with two different correlation methods (Ren & Kuan, 2020) using PBMC RNA sequencing. The TAE calculated by Age_RNAAge showed significant correlations with the chronological age both in PWoH and PWH (*R* > 0.4, *p* < 0.05), being the best fit to our data (Figure S1). Disease status was associated with a poor correlation between biological and chronological age (Holzscheck et al., 2021; Shokhirev & Johnson, 2021). The correlation of TAE with chronological age was higher in PWoH (*R* = 0.62, *p* = 0.0098) than in PWH (*R* = 0.45, *p* < 0.0001) (Figure 1a), indicating a less pronounced correlation in PWH than in negative individuals. To address the aging process's non‐linearity (the aging rate and the manifestation of age‐related changes are not constant over time) (Jylhävä et al., 2017), we calculated the age by subtracting the chronological age from the transcriptomic age. The median (IQR) delta age on PWH was 2.89 (−2.88 to 9.12), indicating high heterogeneity among the PWH. As earlier studies showed that an increase in 5 years of the biological age was associated with an increase in morbidity of around 20% (Chen et al., 2016), we grouped the PWH based on this threshold with an accelerated aging process (AAP, delta age >5, *N* = 75), the regular aging process (RAP, −5 < delta age <5, *N* = 64) and decreasing aging process (DAP, delta age < −5, *N* = 36) groups (Figure 1b). Interestingly, a negative correlation was observed between delta age and chronological age (Figure 1c). PWH under 55 years (*n* = 105) displayed a higher proportion of AAP (*n* = 61/105, 56%) compared to DAP (*n* = 11/105, 10%), while PWH over 55 displayed a higher proportion of DAP (*n* = 26/73, 35%) compared to AAP (*n* = 16/73, 22%) this could be due to the healthy survivor bias. Comparisons of clinical features between aging groups are given in Table 1 and Table S2.

**FIGURE 1:**
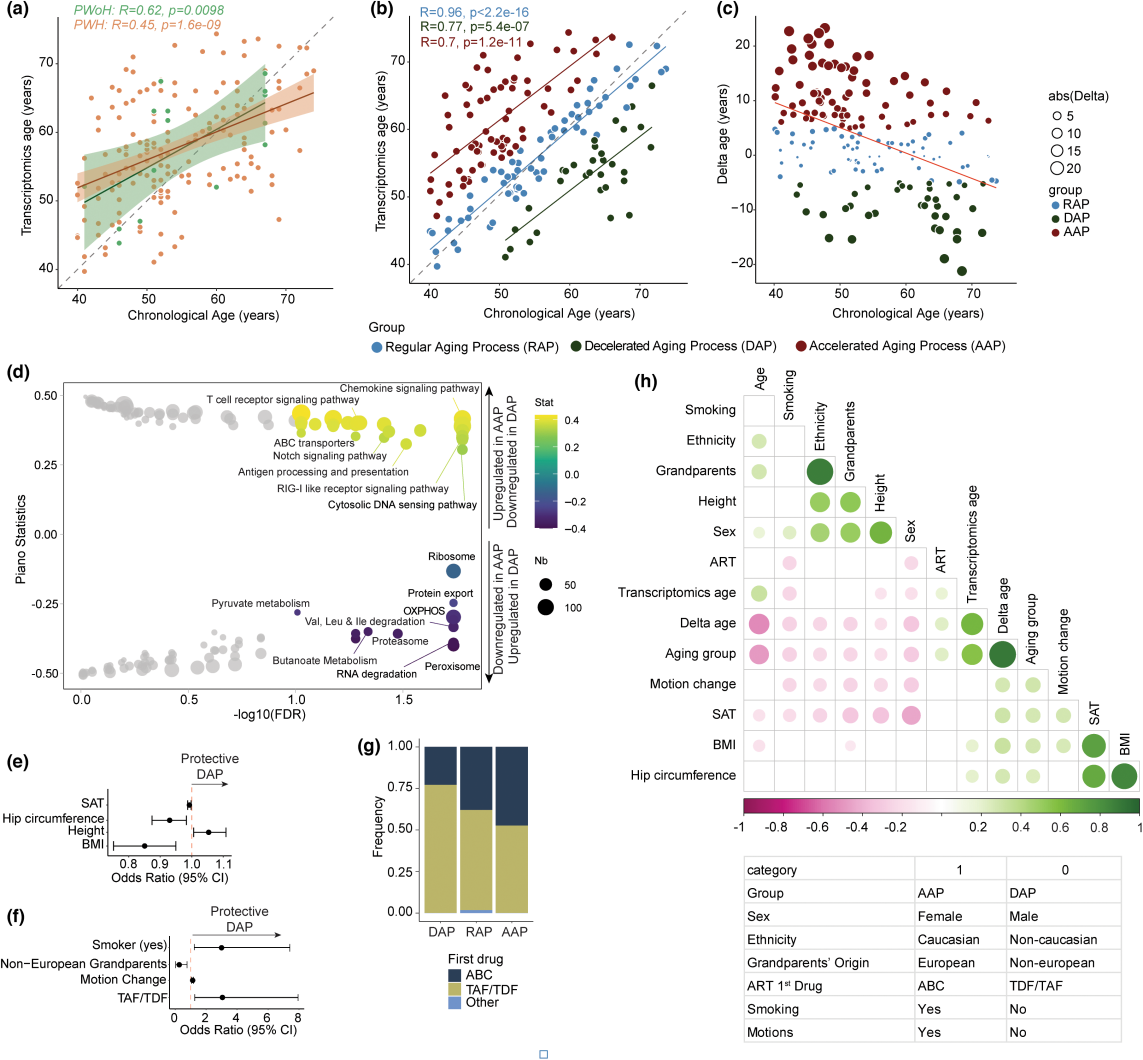
(a) Scatter plot of chronological age (*x*‐axis) versus computed transcriptomics age by RNAAge TAE in PWoH (green) and PWH (orange) (*y*‐axis). Each dot represents one patient. A regression line with a 95% confidence interval, coefficient of correlation (R), and associated *p*‐value (p) was added for each group. The dotted lines represent a perfect positive correlation. **(b)** Scatter plot of chronological age (*x*‐axis) versus computed transcriptomics age by RNAAge TAE in PWH separated into age groups based on delta age (AAP = delta age >5, RAP = −5 < delta age <5, and DAP = delta age < −5) (*y*‐axis). **(c)** Scatter plot of chronological age by RNAAge TAE (*x*‐axis) versus delta age (*y*‐axis) in PWH. Each dot represents a patient. The dot color is based on delta age (*y*‐axis). Size is based on absolute delta age. **(d)** Bubble plot of significant Kyoto Encyclopedia of Genes and Genomes (KEGG) terms enriched in genes differing AAP and DAP [Piano, false discovery rate (FDR <0.1)]. The color was defined based on Piano statistics and size by FDR. **(e)** Forest plot of the univariate logistic regression model to identify the association between the aging group (AAP/DAP) and continuous clinical variables. Odds ratios (OR) and 95% confidence intervals are indicated. **(f)** Forest plot of the univariate logistic regression model to identify the association between the aging group (AAP/DAP) and categorical clinical variables. **(g)** Stacked bar charts representing the frequency of the first ART drug (ABC, TDF/TAF, Other) used depending on the aging groups (AAP, RAP, DAP). **(h)** Correlation plot between clinical, lifestyle, genetics, and aging parameters in PWH. Color represents the Pearson correlation coefficient between two variables.

**TABLE 1:** Patients' clinical, demographic, and lifestyle data.

Parameters	RAP	AAP	DAP	*p*
*N* (%)	64 (36)	77 (43)	37 (21)	
Age in years, median (IQR)	54 (49–62)	49 (46–53)	63 (54–66)	**<0.0001**
Sex, Female, *N* (%)	2 (3)	17 (22)	—	**<0.001**
Ethnicity, Caucasian, *N* (%)	58 (91)	68 (78)	35 (95)	**0.042**
Missing	1 (2)	2 (3)	—	
Grandparents' Origin, Europeans *N* (%)	61 (95)	59 (77)	35 (95)	**0.006**
Missing	—	2 (2.5)	—	
Mode of transmission, *N* (%)				
Homosexual/bisexual	48 (75)	48 (62)	29 (78)	0.443
Heterosexual	13 (20)	21 (27)	9 (16)	
Other/unknown	3 (5)	7 (9)	2 (5)	
Missing	—	1 (1)	—	
CD4 Nadir, cells/mL, median (IQR)	240 (145–300)	250 (121–330)	151 (61–291)	0.259
CD4 at ART Initiation, cells/mL, median (IQR)	283 (159–355)	281 (147–358)	225 (68–350)	0.473
Viral Load at ART initiation, log copies/mL, median (IQR)	4.97 (4.37–5.49)	4.94 (4.31–5.37)	5.19 (3.82–5.61)	0.842
CD4 at sampling, cells/mL, median (IQR)	695 (529–871)	700 (550–870)	660 (580–930)	0.776
CD8 at sampling, cells/mL, median (IQR)	836 (640–1200)	800 (600–944)	740 (566–1200)	0.448
Viral load (<50 copies/mL), *N* (%)	63 (98)	73 (95)	36 (97)	0.216
Duration of treatment in years, median (IQR)	14 (5–17)	14 (6–18)	16 (9–19)	0.206
Current treatment, first drug, *N* (%)				**0.042**
ABC	22 (34)	36 (47)	8 (22)	
TAF/TDF	35 (55)	40 (52)	27 (73)	
Other	1 (2)	—	—	
Missing	6 (9)	1 (1)	2 (5)	
Current treatment, third drug, *N* (%)				**0.042**
INSTI	13 (20)	25 (32)	10 (27)	
NNRTI	30 (62)	30 (39)	14 (38)	
PI	11 (17)	20 (26)	13 (35)	
Missing	—	2 (3)	—	
Previous drug exposure (DDI/AZT/d4t), *N* (%)	41 (64)	50 (65)	27 (73)	0.62
BMI, kg/m^2^, median (IQR)	24.5 (22–27.4)	25.5 (23.4–28.1)	23.7 (21.8–26.2)	**0.029**
Hip circumference in cm, median (IQR)	102 (97–105)	102 (97–108)	99 (94–103)	**0.032**
VAT in cm^2^, median (IQR)	124 (85–177)	94 (52–122)	109 (33–160)	**0.043**
SAT in cm^2^, median (IQR)	109 (80–161)	145 (81–220)	113 (48–135)	**0.03**
Height in meters, median (IQR)	1.8 (1.77–1.84)	1.76 (1.72–1.82)	1.8 (1.76–1.86)	**0.002**
Current smoker, Yes *N* (%)	12 (19)	13 (17)	14 (38)	**0.03**
Bread pieces per day, median (IQR)	4 (2–5)	3 (2–5)	4 (3–5)	**0.02**
Change in motion habits, Yes *N* (%)	20 (31)	31 (40)	5 (14)	**0.016**
Missing	2 (3)	2 (3)	2 (3)	

*Note*: Only relevant significant parameters were presented along with the important HIV‐related parameters. The significant *p*‐values were bold.

Abbreviations: AAP, accelerated aging process; AZT, zidovudine; d4t, stavudine; DAP, decelerated aging process; DDI, didanosine; RAP, regular aging process.

We performed differential gene expression (DGE) and gene set enrichment (GSE) analyses to understand the underlying mechanisms of AAP and DAP. We identified 45 genes dysregulated (33 up and 12 down) in AAP compared to DAP (Table S3). The upregulation of the genes like Fibronectin 1 (FN1) and phospholipase C beta 2 (PLCB2), part of the advanced glycation end‐products (AGEs) and their receptor, RAGE (receptor for AGEs), AGE/RAGE signaling pathway, contribute to the pathogenesis of various age‐related diseases by promoting chronic inflammation, oxidative stress, and cellular damage (Senatus & Schmidt, 2017). A directionally class‐based GSE analysis (Väremo et al., 2013) identified the upregulation of inflammatory pathways like cytokine signaling, cytosolic DNA sensing, and retinoic acid‐inducible gene I (RIG‐I)‐like receptor signaling pathways and immune response like T‐cell receptor signaling, antigen processing, and presentation in AAP. In contrast, metabolic processes like oxidative phosphorylation (OXPHOS), pyruvate metabolism, peroxisome, and proteasome were upregulated in DAP (Figure 1d, Table S4).

We used 143 clinical, demographical, and lifestyle factors from the COCOMO database to identify the confounders potentially responsible or relatively associated with aging acceleration. In AAP PWH, we identified a higher body mass index (BMI) and subcutaneous adipose tissue (SAT), and a lower visceral adipose tissue (VAT) (all *p* < 0.05) compared to DAP. Female sex (22% in AAP compared to 3.1% in RAP vs. none in DAP), PWH from non‐European ancestry (21% in AAP compared to 4.7% in RAP vs. 5.4% in DAP), and use of the abacavir (ABC) (47% in AAP compared to 34% in RAP vs. 22% in DAP) was associated with transcriptomics age acceleration. Interestingly in the PWHs with DAP, there was a higher proportion of current smokers (38%) than in AAP (19%) and RAP (17%) (Table 1).

We performed logistic regression models and interaction analysis to determine associations and report potential interaction or mediation effects between clinical parameters on the accelerated or decelerated aging process. A univariate logistic regression with the binary‐dependent variable being the aging group (AAP/DAP) and each of the significant (*p* < 0.05) clinical parameters as the independent variable identified eight significant clinical parameters of interest. It included metabolic features SAT, BMI, hip circumference (high in AAP), genetic features including height (high in AAP), motions (low in AAP), and origin (more non‐European in AAP), lifestyle including smoking (more in DAP), and finally, HIV‐related parameter ART drug [more tenofovir alafenamide (TAF)/tenofovir disoproxil fumarate (TDF) in DAP) (Table S5) (Figure 1e,f). The proportion of participants using abacavir (ABC) at the time of sampling was significantly higher in AAP (Figure 1g). To identify associations between clinical parameters, we performed a Chi‐square test of independence between categorical variables (Table S6) and Pearson correlations between continuous variables (Table S7). To compare categorical and continuous variables, categorical variables of interest were converted into binary categories and then 0 and 1 values to correlate with continuous values, as indicated in Figure 1h. We found a clear association between sex, ART, and smoking which could explain the differences in biological aging observed between PWH (Figure 1h). The high intercorrelation between parameters suggested interactions or confounding effects. To identify the hierarchy of the associations, we tested all these parameters for interactions and mediation effects. Still, we have not found any significant relationship (Table S8 and Figure S2), probably due to the limited number of samples.

Here, we presented an extensive application of a trained transcriptomics clock and clinical, lifestyle, and demographic data from PWH to define the pace of the biological aging process and potential mechanisms and factors linked to accelerated aging in long‐term treated PWH. The AAP was more prominent among the younger PWH, females, non‐European ethnicity, and the PWHs on abacavir, while DAP was more frequent in the older PWH. An earlier study reported that biological age acceleration quantified on young people might help to prevent age‐related diseases (Belsky et al., 2015). This is valid for the PWH on long‐term treatment. The clinical factors related to obesity in young PWH may reflect the biological aging linked with poor metabolic processes and increased inflammation, as observed in the GSA. In contrast, the DAP in the older PWH could be due to better survival due to genetics, environment, and comorbidities. Interestingly, the only HIV‐related factor associated with AAP was the current use of ABC, which indicates that the previous use of toxic drugs (e.g., didanosine, zidovudine, or stavudine) had a low impact on accelerated biological aging. It is important to note that biological age acceleration is not a definitive measure of health or longevity, but better at predicting mortality than chronological age (Levine, 2013) and might allow identifying the accentuated aging at an earlier time point and be able to develop intervention strategies to slow down or prevent the accelerated aging process and age‐related diseases (Divo et al., 2014).

As PWH presents a complex aging process and is confounded by the ART, sex, genetics, environment, lifestyle, and comorbidities (Akusjarvi & Neogi, 2023), recent studies reported epigenetic age acceleration in PWH (Breen et al., 2022; Esteban‐Cantos et al., 2021) limited by those confounders observed in our study. Though our study is limited by its cross‐sectional study design and smaller samples, the strength of our study is the inclusion of 143 demographic, clinical, HIV‐related, and lifestyle parameters and high intercorrelation between parameters, suggested interactions, or confounding effects. Moreover, our recent multi‐omics study identified complex diseases' state‐omics phenotypes within PWH that a single omics or clinical feature could not explain (Mikaeloff et al., 2023).

To the best of our knowledge, this is the first study to show the biological age acceleration by using the transcriptomics aging clock within PWH, which showed that lifestyle, poor metabolic process, inflammatory status, and use of HIV medication abacavir are associated with the accelerated aging process confounded by the sex and ethnicity. It can be reversed or slowed down by identifying the biological age acceleration in the middle‐age adult PWH and applying personalized medicine and lifestyle intervention to improve their dysregulated metabolic traits, aiming to achieve healthier aging.

## AUTHOR CONTRIBUTIONS

U.N. conceptualized the project. U.N. and S.D.N acquired the funding. M.G., A.D.N., J.H., T.B., J.P.M., S.D.N., and U.N. provided resources for the study. S.D.N. and U.N. supervised the study. F.M., and U.N. developed and designed the methodology. F.M., A.E., M.G., A.D.N., and J.H. carried out the experiments. F.M. performed the formal data analysis, and F.M. and U.N. visualized the results. F.M. and U.N. wrote the original draft of the manuscript. A.E., M.G., A.D.N., J.H., T.B., J.P.M., and S.D.N. reviewed and edited the manuscript. All authors have reviewed and approved the final version.

## CONFLICT OF INTEREST STATEMENT

The authors declare no competing interests.

### OPEN RESEARCH BADGES

This article has earned an Open Data badge for making publicly available the digitally‐shareable data necessary to reproduce the reported results. The data is available in The raw RNAseq data have been deposited in the NCBI/SRA with PRJNA983231.

## Supporting information


Data S1.

## Data Availability

The raw RNAseq data have been deposited in the NCBI/SRA with PRJNA983231. The clinical, lifestyle, and demographic data is confined to the study group, but the request for data can be submitted to cocomo.rigshospitalet@regionh.dk.
