# Supplementary material for: Transcriptomics age acceleration in prolonged treated HIV infection
Source: Aging Cell. 2023 Aug 7;22(10):e13951. doi: 10.1111/acel.13951 (PMC10577541; doi:10.1111/acel.13951)
Supplement: Supplementary file 1 — Data S1. [file ACEL-22-e13951-s001.pdf]

Supplementary Material

Extended Methods

PWH Cohort and Clinical Data: The patient population (n=178) included in this study was part of the larger COCOMO cohort (n=1099) of PWH. Extensive clinical, demographical, diet, and lifestyle data (n=148) were collected from the COCOMO database. Additional 18 self-reported healthy people without HIV (PWoH) from the Danish populations with similar sex proportion (89% male, 16/18), median BMI (IQR) of 26(25-29), and median age (IQR) of 56 (51-67) years were used to identify the best transcriptomics age estimation (TAE) suited for the population.

Genome-wide RNA sequencing (RNAseq): The genome-wide RNAseq has been performed on the peripheral blood mononuclear cells (PBMC). Total RNA was extracted using Quick-RNA™ Miniprep Plus Kit (Zymo Research, USA). The library was prepared with Illumina TruSeq Stranded mRNA (Illumina, USA) with Poly-A selection followed by sequencing on NovaSeq6000 (NovaSeq Control Software 1.7.5/RTA v3.4.4) using 'NovaSeqXp' workflow in 'S4' mode flowcell. The RNAseq data were processed with the nf-core/rnaseq (Ewels et al., 2020).

Transcriptomics age estimation (TAE): Four transcriptomics clocks trained on large cohorts of healthy patients were selected: BitAge(Meyer & Schumacher, 2021), BURNS(Shokhirev & Johnson, 2021), and RNAAgeCalc, with two different methodologies(Ren & Kuan, 2020). The biological age of each patient was calculated based on their transcriptomics data using the available R package RNAAgeCalc, python script (https://github.com/Meyer-DH/AgingClock), or web user interface (http://burns.salk.edu/). The transcriptomics age for each clock was plotted against the chronological age for healthy and PWH. Pearson correlation and linear regression between transcriptomics age and chronological age were performed for each clock. The clock with the best results in healthy controls was taken for further analysis. Delta was calculated between chronological and transcriptomics ages (delta age=Transcriptomics age-Chronological Age). Patients were separated into three groups decelerated aging process (DAP: Delta age>-5), the regular aging process (RAP:-5<Delta age<5 and the accelerated aging process (AAP, Delta age>5). Differential expression analysis was performed between groups using the R package DESeq2 (Love, Huber, & Anders, 2014). The p-values were adjusted using Benjamini-Hochberg (BH) corrections, and the cutoff was set at 0.1. Pathway analysis was performed using the R package Piano(Väremo, Nielsen, & Nookaew, 2013), and Kyoto Encyclopedia of Genes and Genomes (KEGG) terms with false discovery rate (FDR)<0.1 was kept as significant.

Statistics analysis: Clinical data were filtered for missing values and clinical features, with more than 30% of missing values removed. Mann-Whitney U and Kruskal-W–Wallis H were used to compare continuous variables pairwise and between several groups. Discrete variables were compared between groups with the Chi-Square Test if the expected values of the contingency table were five or more; otherwise, Fisher's Exact Test was applied. Categorical data were regrouped into two categories for Transmission mode, first ART regimen, demography (Ethnicity, Grandparents' origin. Logistic regression was performed using the glm function from stats R package and parameters (http://www.R-project.org/). The cutoff for the p-value was set to 0.05. Interactions between clinical parameters were tested using R packages effects and emmeans (https://github.com/rvlenth/emmeans). Categorical values were converted into 0 and 1 values and used for Pearson correlations with all clinical values computed. Correlations with p-values under 0.1 were considered significant.

Visualization methods: Barplots, bubble plots, forest plots, and scatters plots were made using R package ggplot2 (Wickham, 2009). The correlation plot was done using R package corrplotv0.92 (https://github.com/taiyun/corrplot.).

References

Ewels, P. A., Peltzer, A., Fillinger, S., Patel, H., Alneberg, J., Wilm, A., . . . Nahnsen, S. (2020). The nf-core framework for community-curated bioinformatics pipelines. *Nat Biotechnol, 38*(3), 276-278. doi:10.1038/s41587-020-0439-x

Love, M. I., Huber, W., & Anders, S. (2014). Moderated estimation of fold change and dispersion for RNA-seq data with DESeq2. *Genome Biol, 15*(12), 550. doi:10.1186/s13059-014-0550-8

Meyer, D. H., & Schumacher, B. (2021). BiT age: A transcriptome-based aging clock near the theoretical limit of accuracy. *Aging Cell, 20*(3), e13320. doi:10.1111/acel.13320

Ren, X., & Kuan, P. F. (2020). RNAAgeCalc: A multi-tissue transcriptional age calculator. *PLoS One, 15*(8), e0237006. doi:10.1371/journal.pone.0237006

Shokhirev, M. N., & Johnson, A. A. (2021). Modeling the human aging transcriptome across tissues, health status, and sex. *Aging Cell, 20*(1), e13280. doi:10.1111/acel.13280

Väremo, L., Nielsen, J., & Nookaew, I. (2013). Enriching the gene set analysis of genome-wide data by incorporating directionality of gene expression and combining statistical hypotheses and methods. *Nucleic Acids Res, 41*(8), 4378-4391. doi:10.1093/nar/gkt111

Wickham, H. (2009). ggplot2: elegant graphics for data analysis New York. *NY: Springer*.

Supplementary figures

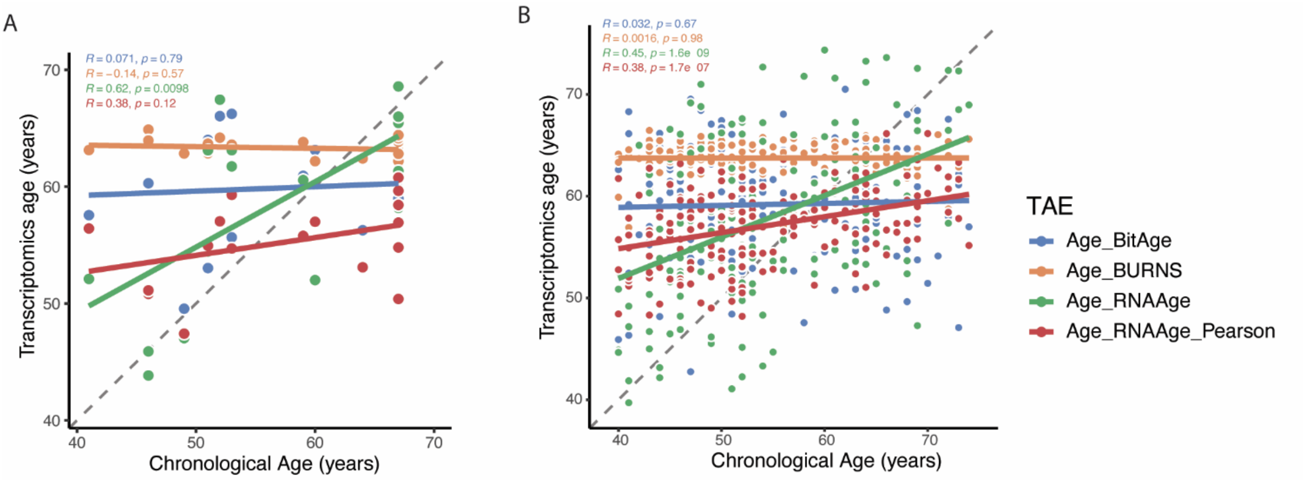

Figure S1: Scatter plot of the chronological age of healthy controls (A) and HIV patients (B) versus the transcriptomics age calculated by four TAE (BitAge, BURNS, RNAAge, and RNAAge Pearson). The coefficient of correlation and associated p-value and regression line were added for each clock.

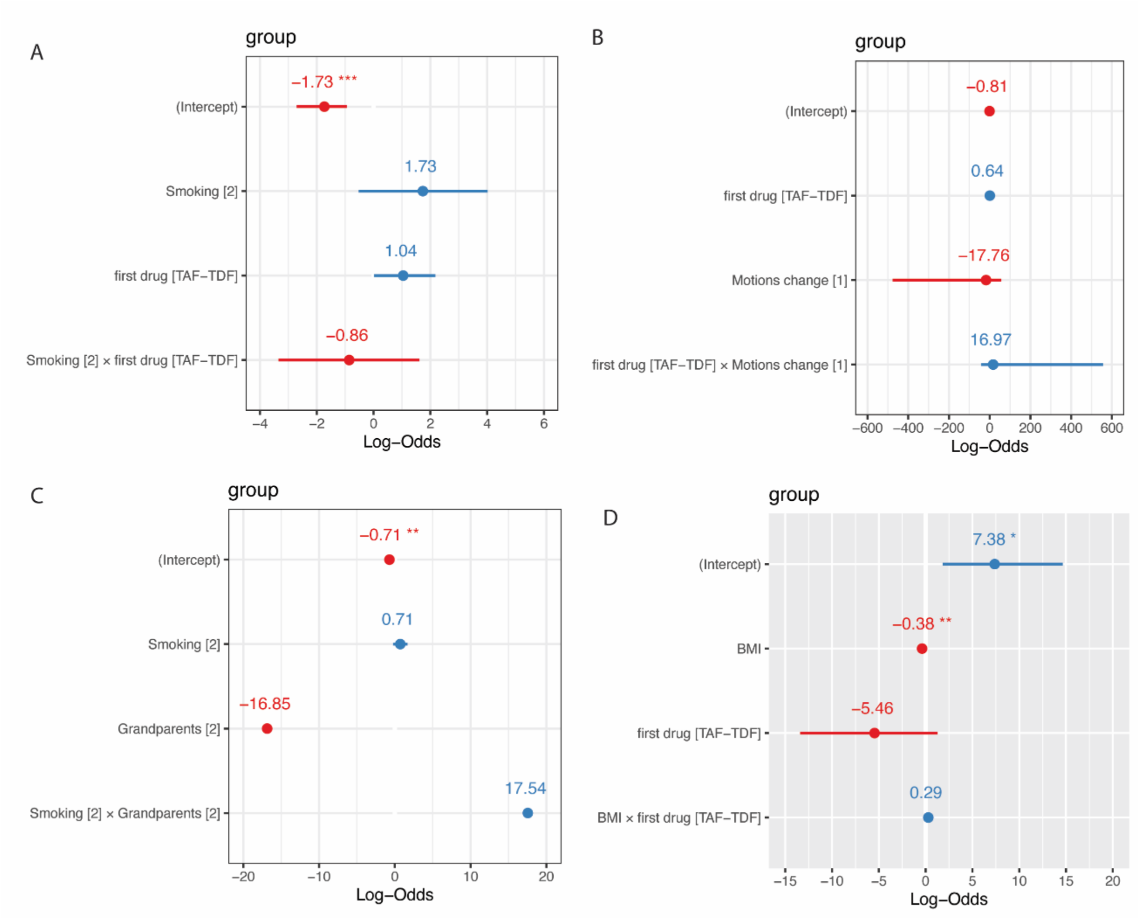

Figure S2: Forest plots representing the odd ratios from logistic regression analysis. Interactions are tested. Significant interactions ($p<0.05$) are indicated by stars.

Table S1. The clinical features of the complete cohort and the sub cohort

	Complete cohort	Subcohort
**N**	1098	178
**Age in years, Median (IQR)**	50.11(42.78-58.02)	52.4 (47.17-62.23)
**Sex, Male, N (%)**	936 (85.25)	159 (89.33)
**Ethnicity Caucasian, N (%)**	946(88)	153(87)
**Mode of transmission, N (%)**		
**Homosexual/bisexual**	774 (71)	125 (71)
**Heterosexual**	235 (22)	40 (23)
**Other/unknown**	80 (7.35)	12 (6.78)
**CD4 Nadir, cells/mL, Median (IQR)**	235(117.5-350)	237(108-320)
**CD4 at ART Initiation, cells/mL, Median (IQR)**	260(140-398)	272 (132-359)
**Viral Load at ART initiation, log copies/mL, Median (IQR)**	4.86 (4.29-5.38)	4.99 (4.31-5.48)
**CD4 at sampling, cells/mL, Median (IQR)**	690 (520-890)	693 (550-881)
**CD8 at sampling, cells/mL, Median (IQR)**	840 (630-1190)	808 (600-1100)
**CD4/CD8 ratio**	0.81 (0.57-1.13)	0.84(0.6-1.21)
**Viral load (<50 copies/mL), N (%)**	1030 (94.67%)	172 (97.18%)
**Duration of treatment in years, median (IQR)**	11 (5 -17)	14 (7-18)
**Current Treatment, 1st drug, N (%)**		
**ABC**	270 (24.5)	66 (37.1)
**TDF/TAF**	750 (68.3)	102 (57.3)
**Other**	4 (0.36)	1(0.56)
**Missing**	74 (6.74)	9 (5.06)
**Current Treatment, 3rd drug, N (%)**		
**NNRTI**	479 (43.6)	84 (47.19)
**PI/r**	379 (34.5)	44 (24.72)
**INSTI**	238 (21.67)	48 (26.97)
**Missing**	2 (0.1)	2 (1.12)
**Previous exposure to ddi/d4t/AZT, N (%)**		
**BMI, kg/m2, Median (IQR)**	24.6 (22.4-27.1)	24.7 (22.1-27.3)
**Smoking status**		
**Never smoked**	369 (33.6)	75 (42.1)
**Ex smoker**	374 (34.1)	64 (36)
**Current smoker**	316 (28.8)	39 (21.9)
**Unknown**	39 (3.6)	0

Table S2: Reported p values from Statistics of clinical parameters. FET: Fisher's Exact Test, WST: Wilcoxon signed-rank test, MW: Mann Whitney U test, χ^2 : Chi-square test. The choice of the test was based on the normal distribution of the data

Name	P (3 groups)	Test (3 groups)	P (2 groups)	Test (2 groups)
Gender (male/female)	0.0005	FET	0.0048	χ^2
Motion habits changed markedly past 1 year (yes/no)	0.0135	FET	0.0025	FET
Number of years with Daily use of television (Years)	0.0003	WST	0.0001	MW
Adulthood (after age 18) exposure of passive smoking (Years)	0.0153	WST	0.0063	MW
Age (years)	0.0000	WST	0.0000	MW
Body mass index (kg/m2)	0.0278	WST	0.0116	MW
Body mass index categorical (underweight, normal, overweight, obese)	0.0395	FET	0.0365	FET
Bread, pieces per day (Number per day)	0.0199	WST	0.0191	MW
Childhood (before age 18) exposure of passive smoking (Years)	0.0241	WST	0.0078	MW
Current smoker (yes/no)	0.0302	χ^2	0.0258	χ^2
First ART drug (ABC, TDF-TAF, Other)	0.0440	FET	0.0215	FET
Height (m)	0.0021	WST	0.0247	MW
Hip circumference (cm)	0.0317	WST	0.0127	MW
subcutaneous adipose tissue	0.0301	WST	0.0162	MW
Ethnicity (Caucasian, non-caucasian)	0.0405	χ^2	0.0810	χ^2
Grandparents origin (European, non European)	0.0080	FET	0.0605	FET
Medication for eye conditons (yes/no)	0.0430	FET	0.0925	FET
Saturation (%)	0.0156	WST	0.3398	MW
Third ART drug (PI, INSTI, NNRTI)	0.0415	χ^2	0.6226	χ^2
Viceral adipose tissue (VAT)	0.0437	WST	0.7074	MW
Coke light consumption (bottles of 0.5 L per week)	0.0803	WST	0.0375	MW
Current smoking status (Never, Current, Ex-smoker, Unknown)	0.0501	χ^2	0.0190	χ^2
Frequency of consumption of > 5 drinks in one occasion during the past year (Never, 1< per month, 1-3 per month, 1 per week, > 1 per week)	0.1429	FET	0.0470	FET
Hypertension (yes/no)	0.0548	χ^2	0.0336	χ^2
Systolic blood pressure, left arm (mm mercury)	0.1224	WST	0.0295	MW
White wine (Never, Monthly, Weekly, Daily)	0.0560	FET	0.0115	FET
Alaninaminotransferase	0.2949	WST	0.1240	MW
Alcohol (Unit:gram / per week)	0.9991	WST	0.9575	MW
All alcoholic drinks (Never, Monthly, Weekly, Daily)	0.8206	FET	0.5262	FET
Ankle bracial index (> 0.9, < 0.9)	0.0605	FET	0.1114	FET
Anti dyslipedemics (yes/no)	0.2224	FET	0.4698	FET
Anti-coagulative therapy (yes/no)	0.2744	FET	0.3008	FET
Antihypertensives (yes/no)	0.1767	χ^2	0.8156	χ^2

Anxiolytics/antidepressant (yes/no)	0.3273	FET	0.2824	FET
Arthritis medication (yes/no)	0.1049	FET	0.2919	FET
Aspirin (yes/no)	0.8951	FET	0.6302	FET
Beef intake (Times per week in average)	0.3310	WST	0.1403	MW
Beer (Never, Monthly, Weekly, Daily)	0.9660	FET	0.8291	FET
Bloodpressure, left leg (mm mercury)	0.7140	WST	0.4572	MW
Bloodpressure, right leg (mm mercury)	0.8099	WST	0.6664	MW
Candy intake (Never, 1-3 per month, 1-2 per week, 3-4 per week, 1 per day, > 1 per day)	0.8461	FET	0.6132	FET
CD4 at ART initiation	0.4731	WST	0.2645	MW
CD4/CD8 ratio	0.1573	WST	0.4353	MW
Central obesity (yes/no)	0.3642	χ^2	0.2342	χ^2
Cheese Cold cut (Times per week in average)	0.2128	WST	0.1093	MW
Chocolate intake (Never, 1-3 per month, 1-2 per week, 3-4 per week, 1 per day, > 1 per day)	0.9020	FET	0.9555	FET
Choice of fat for preparing warm dishes (None, Butter, Spreadable, Shortening, Margarine, Olive oil, Other oil, Other)	0.8656	FET	0.5997	FET
CMV IgG (yes/no)	0.3138	FET	0.8576	FET
Coffe consumption (cup per week)	0.2524	WST	0.4635	MW
Coke consumption (bottles of 0.5 L per week)	0.8243	WST	0.6840	MW
Current CD4 count	0.7759	WST	0.6006	MW
Current CD8 count	0.4477	WST	0.7480	MW
Current/previous marihuana use (yes/no)	0.4608	FET	0.2904	FET
Daily use of Computer (Minutes)	0.8802	WST	0.6926	MW
Daily use of Computer games (Minutes)	0.3118	WST	0.1426	MW
Daily use of mobile phone (Minutes)	0.7476	WST	0.4983	MW
Daily use of television (Minutes)	0.2715	WST	0.7525	MW
Days per month with nightly work (Days)	0.5848	WST	0.7655	MW
Deli meats Cold cut (Times per week in average)	0.8712	WST	0.8508	MW
Diabetes (yes/no)	0.6357	FET	0.6621	FET
Diastolic blood pressure, left arm (mm mercury)	0.1787	WST	0.3176	MW
Diastolic blood pressure, right arm (mm mercury)	0.1336	WST	0.2149	MW
Diuretics (yes/no)	0.4213	FET	0.5462	FET
Drugs to treat heart conditions (yes/no)	0.6152	FET	1.0000	FET
Duration ART(years)	0.2060	WST	0.3395	MW
Duration since last meal (0-1, 1-2, 2-3, 3-4, 4-5, 5-6, 6-7, 7-8, >8)	0.3053	FET	0.1684	FET
D-vitamin (yes/no)	0.7931	FET	1.0000	FET
E-cigarettes (no / yes with nicotine / yes without nicotine)	0.5137	FET	0.5532	FET
Ecological food and drinks (%)	0.5918	WST	0.5584	MW
eGFR(mL/min/1.73m2)	0.2669	WST	0.1220	MW
Fastfood intake (Times per week in average)	0.4797	WST	0.2196	MW
Fish Cold cut (Times per week in average)	0.9906	WST	0.8724	MW
Fish intake (Times per week in average)	0.5833	WST	0.5140	MW

Fruit intake (Never, 1-3 per month, 1-2 per week, 3-4 per week, 5-6 per week, 1 per day , 2-3 per day, >3 per day)	0.6517	FET	0.3508	FET
Hours outdoor (Hours)	0.1266	WST	0.3909	MW
Hypnotica (yes/no)	0.2414	FET	0.1819	FET
Juice consumption (glass per week)	0.2878	WST	0.1570	MW
Lamb intake (Times per week in average)	0.4946	WST	0.5201	MW
Limonade consumption (glass per week)	0.0792	WST	0.0559	MW
Liquor (Never, Monthly, Weekly, Daily)	0.8101	FET	0.9795	FET
Liver stiffness measurement (kpa)	0.2980	WST	0.7107	MW
Log10 VL at ART initiation	0.8425	WST	0.9033	MW
Low fat milk consumption (glass per week)	0.3565	WST	0.4759	MW
Lowest recorded CD4	0.2588	WST	0.1249	MW
Main courses (Number per day)	0.7391	FET	0.6982	FET
Metabolic Syndrome (yes/no)	0.5048	χ^2	0.5961	χ^2
Mode of HIV transmission (MSM, Heterosexual, Other)	0.4683	FET	0.2619	FET
Natural remedies (yes/no)	0.7071	FET	0.6452	FET
Nicotine substitution(yes/no)	0.4108	FET	1.0000	FET
Number of years with Daily use of Computer (Years)	0.8204	WST	0.5197	MW
Number of years with Daily use of Computer games (Years)	0.5941	WST	0.5260	MW
Number of years with Daily use of mobile phone (Years)	0.6989	WST	0.3639	MW
Origin defined as in the Danish HIV cohort (European, non European)	0.1010	χ^2	0.0768	χ^2
Other alcohlic drinks(Never, Monthly, Weekly, Daily)	0.6052	FET	0.8286	FET
Other antidiabetic medication (yes/no)	0.1919	FET	0.1724	FET
Other medication (yes/no)	0.7136	FET	0.8291	FET
Other natural remedies (yes/no)	0.6037	FET	1.0000	FET
Other pain relief medication (yes/no)	0.6117	FET	1.0000	FET
Other vitamin (yes/no)	0.8556	FET	0.8381	FET
Paracetamol (yes/no)	0.5547	FET	0.3868	FET
Passive smoking exposure (Hours per day)	0.5746	WST	0.5603	MW
Pate Cold cut (Times per week in average)	0.0665	WST	0.0539	MW
P-Cholesterol+ester, i HDL (mmol/L)	0.8439	WST	0.7210	MW
P-Creatininium (µmol/L)	0.5076	WST	0.2733	MW
Physical activity in spare time (Inactive, Slightly active, Moderated active, Very active)	0.8781	FET	0.7451	FET
P-Kolesterol LDL (mmol/L)	0.1032	WST	0.7737	MW
Pork intake (Times per week in average)	0.1954	WST	0.0698	MW
Positive ab anti-HBs (yes/no)	0.2549	FET	0.3023	FET
Positive HBsAg (yes/no)	0.1909	FET	0.5577	FET
Positive HCV antibodies (yes/no)	0.1414	FET	0.7446	FET
Positive HCV RNA (yes/no)	0.1129	FET	0.1734	FET
Positive IgG anti-HBc (yes/no)	0.4803	FET	0.4968	FET
Poultry intake (Times per week in average)	0.1232	WST	0.3012	MW

Previous AIDS defining condition (yes/no)	0.3786	χ^2	0.7182	χ^2
Previous ddi/AZT/d4t ART exposure	0.6235	χ^2	0.5192	χ^2
Previous smoker (yes/no)	0.2869	FET	0.1264	FET
P-Triglycerid (mmol/L)	0.9421	WST	0.9397	MW
Pulse (beats per minute)	0.5762	WST	0.9437	MW
Red wine (Never, Monthly, Weekly, Daily)	0.6087	FET	0.6197	FET
Skim milk consumption (glass per week)	0.9996	WST	0.9898	MW
Soda consumption (bottles of 0.5 L per week)	0.9414	WST	0.8692	MW
Soda light consumption (bottles of 0.5 L per week)	0.4920	WST	0.2389	MW
Syphilis infection (yes/no)	0.9095	FET	0.8106	FET
Systolic blood pressure, right arm (mm mercury)	0.0812	WST	0.0505	MW
Tea consumption (cup per week)	0.1904	WST	0.4536	MW
time from awakening to first cup of coffee (Hours)	0.3230	WST	0.3996	MW
Transmission origin (Europe, Non Europe)	0.0808	χ^2	0.7089	χ^2
Treatment for hypothyroidism (yes/no)	1.0000	FET	1.0000	FET
Tx for asthma/bronchitis/COPD (yes/no)	0.5802	FET	0.5367	FET
Tx for gastric acidity (yes/no)	0.1629	FET	0.6697	FET
Type of butter on bread (None, Butter, Spreadable, Margarine, Other)	0.4028	FET	0.6477	FET
Type of chocolate (Dark chocolate, Other)	0.3303	FET	0.3003	FET
Vegetable intake (Never, 1-3 per month, 1-2 per week, 3-4 per week, 5-6 per week, 1 per day , 2-3 per day, >3 per day)	0.0755	FET	0.3918	FET
Viral load (<50, >50)	0.2334	FET	1.0000	FET
Waist circumference (cm)	0.5945	WST	0.4223	MW
Waist hip ratio (ratio)	0.4378	WST	0.2720	MW
Water consumption (bottles of 0.5 L per week)	0.7227	WST	0.5280	MW
Weekly consumption (beer) (Number of 33 cl)	0.9628	WST	0.9381	MW
Weekly consumption (Liquor) (Number of 33 cl)	0.5105	WST	0.7090	MW
Weekly consumption (Red wine) (Number of 33 cl)	0.8260	WST	0.8239	MW
Weekly consumption (White wine) (Number of 33 cl)	0.3902	WST	0.5671	MW
Weight of participant (kg)	0.3037	WST	0.1811	MW
Whole milk consumption (glass per week)	0.5296	WST	0.6621	MW

Table S3 : Differential expression analysis between AAP and DAP (DESeq2, FDR < 0.1)

Transcript	Gene Name	LogFC	p	FDR
ENSG00000073282	TP63	-0.556	0.000	0.094
ENSG00000099994	SUSD2	0.557	0.000	0.094
ENSG00000100991	TRPC4AP	0.138	0.000	0.094
ENSG00000102055	PPP1R2C	6.868	0.000	0.017
ENSG00000103335	PIEZO1	0.261	0.000	0.063
ENSG00000105609	LILRB5	1.681	0.000	0.000
ENSG00000107331	ABCA2	0.363	0.000	0.064
ENSG00000107862	GBF1	0.199	0.000	0.094
ENSG00000108039	XPNPEP1	0.144	0.000	0.041
ENSG00000115112	TFCP2L1	0.796	0.000	0.093
ENSG00000115414	FN1	1.431	0.000	0.076
ENSG00000125434	SLC25A35	0.228	0.000	0.045
ENSG00000126012	KDM5C	0.310	0.000	0.001
ENSG00000130703	OSBPL2	0.115	0.000	0.070
ENSG00000130940	CASZ1	0.328	0.000	0.005
ENSG00000134369	NAV1	0.273	0.000	0.094
ENSG00000134765	DSC1	-0.820	0.000	0.094
ENSG00000137841	PLCB2	0.230	0.000	0.058
ENSG00000141503	MINK1	0.212	0.000	0.080
ENSG00000144711	IQSEC1	0.156	0.000	0.064
ENSG00000145016	RUBCN	0.120	0.000	0.094
ENSG00000147050	KDM6A	0.315	0.000	0.056
ENSG00000151692	RNF144A	-0.234	0.000	0.094
ENSG00000154165	GPR15	-1.024	0.000	0.074
ENSG00000158270	COLEC12	-0.973	0.000	0.009
ENSG00000160299	PCNT	0.173	0.000	0.092
ENSG00000160305	DIP2A	0.342	0.000	0.005
ENSG00000162627	SNX7	1.555	0.000	0.018
ENSG00000162747	FCGR3B	0.917	0.000	0.045
ENSG00000167978	SRRM2	0.239	0.000	0.074
ENSG00000169247	SH3TC2	0.753	0.000	0.093
ENSG00000171490	RSL1D1	-0.156	0.000	0.080
ENSG00000173114	LRRN3	-1.189	0.000	0.002
ENSG00000175061	SNHG29	-0.239	0.000	0.043
ENSG00000175544	CABP4	0.483	0.000	0.045
ENSG00000184368	MAP7D2	2.536	0.000	0.000
ENSG00000186871	ERCC6L	1.239	0.000	0.005
ENSG00000188827	SLX4	0.258	0.000	0.040
ENSG00000197136	PCNX3	0.223	0.000	0.094
ENSG00000204650	LINC02210	-0.299	0.000	0.054
ENSG00000214113	LYRM4	-0.250	0.000	0.062
ENSG00000214401	KANSL1-AS1	-0.678	0.000	0.058
ENSG00000227165	WDR11-AS1	0.706	0.000	0.086
ENSG00000229807	XIST	10.109	0.000	0.000
ENSG00000230006	ANKRD36BP2	-0.942	0.000	0.043

Table S4 : Gene set enrichment analysis using KEGG metabolism terms and based on differential gene expression between AAP and DAP (PIANO, FDR<0.1)

Name	Distinct Direction Up.	FDR	No genes Up	Distinct Direction Down	FDR	No Genes Down
CYTOSOLIC DNA SENSING PATHWAY	0.305	0.017	34	0.695	1.000	10
RIG I LIKE RECEPTOR SIGNALING PATHWAY	0.340	0.017	42	0.660	1.000	16
B CELL RECEPTOR SIGNALING PATHWAY	0.347	0.017	53	0.653	1.000	20
CHEMOKINE SIGNALING PATHWAY	0.394	0.017	118	0.606	1.000	61
ANTIGEN PROCESSING AND PRESENTATION	0.373	0.026	49	0.627	1.000	20
PHOSPHATIDYLINOSITOL SIGNALING SYSTEM	0.354	0.017	50	0.646	1.000	22
PRIMARY IMMUNODEFICIENCY	0.364	0.094	23	0.636	1.000	11
FC EPSILON RI SIGNALING PATHWAY	0.370	0.037	52	0.630	1.000	22
NOTCH SIGNALING PATHWAY	0.348	0.039	32	0.652	1.000	14
FC GAMMA R MEDIATED PHAGOCYTOSIS	0.373	0.017	63	0.627	1.000	29
VEGF SIGNALING PATHWAY	0.375	0.026	47	0.625	1.000	22
COMPLEMENT AND COAGULATION CASCADES	0.388	0.067	40	0.612	1.000	18
REGULATION OF ACTIN CYTOSKELETON	0.389	0.017	125	0.611	1.000	71
ADIPOCYTOKINE SIGNALING PATHWAY	0.393	0.094	42	0.607	1.000	18
T CELL RECEPTOR SIGNALING PATHWAY	0.404	0.050	64	0.596	1.000	41
ENDOCYTOSIS	0.394	0.017	114	0.606	1.000	66
ECM RECEPTOR INTERACTION	0.394	0.067	57	0.606	1.000	25
ABC TRANSPORTERS	0.353	0.053	26	0.647	1.000	12
NATURAL KILLER CELL MEDIATED CYTOTOXICITY	0.396	0.039	78	0.604	1.000	40
GAP JUNCTION	0.396	0.081	53	0.604	1.000	29
JAK STAT SIGNALING PATHWAY	0.401	0.051	85	0.599	1.000	48
LEUKOCYTE TRANSENDOTHELIAL MIGRATION	0.401	0.057	69	0.599	1.000	37
AXON GUIDANCE	0.401	0.050	79	0.599	1.000	41
ERBB SIGNALING PATHWAY	0.403	0.094	56	0.597	1.000	29
VASCULAR SMOOTH MUSCLE CONTRACTION	0.404	0.051	63	0.596	1.000	35
TOLL LIKE RECEPTOR SIGNALING PATHWAY	0.395	0.053	62	0.605	1.000	29
NEUROACTIVE LIGAND RECEPTOR INTERACTION	0.416	0.067	128	0.584	1.000	60
FOCAL ADHESION	0.416	0.017	121	0.584	1.000	72

CALCIUM SIGNALING PATHWAY	0.418	0.067	94	0.582	1.000	58
MAPK SIGNALING PATHWAY	0.435	0.094	143	0.565	1.000	95
PEROXISOME	0.600	1.000	30	0.400	0.018	46
RNA DEGRADATION	0.609	1.000	23	0.391	0.018	34
PYRUVATE METABOLISM	0.624	1.000	14	0.376	0.053	24
PROTEASOME	0.642	1.000	16	0.358	0.033	28
FATTY ACID METABOLISM	0.643	1.000	11	0.357	0.053	22
BUTANOATE METABOLISM	0.650	1.000	8	0.350	0.046	21
VALINE LEUCINE AND ISOLEUCINE DEGRADATION	0.667	1.000	12	0.333	0.018	31
PROPANOATE METABOLISM	0.693	1.000	8	0.307	0.018	24
OXIDATIVE PHOSPHORYLATION	0.702	1.000	26	0.298	0.018	84
LIMONENE AND PINENE DEGRADATION	0.719	1.000	1	0.281	0.098	9
PROTEIN EXPORT	0.753	1.000	6	0.247	0.018	17
RIBOSOME	0.868	1.000	4	0.132	0.018	81

Table S5 : Univariate Logistic regression AAP/DAP and clinical parameters

Parameter	Odd Ratio	p	CI_Low	CI_high
Transcriptomics age	0.8160	0.0000	0.7401	0.8799
Body mass index (kg/m2)	0.8509	0.0062	0.7521	0.9495
Ethnicity (Caucasian, non-caucasian)	4.3750	0.0592	1.1439	28.8021
first_drug_TAF-TDF	3.0375	0.0165	1.2670	7.9377
SAT	0.9929	0.0085	0.9873	0.9979
AGE	1.1473	0.0000	1.0883	1.2176
Grandparents origin (European, non European)	0.2008	0.0401	0.0305	0.7667
Height of participant (cm)	1.0537	0.0323	1.0063	1.1084
Hip circumference (cm)	0.9301	0.0148	0.8743	0.9831
Current smoker (yes/no)	2.9967	0.0160	1.2292	7.4163
Motion habits changed markedly past 1 year (yes/no)	0.2289	0.0059	0.0719	0.6107

Table S6 : Chi-Square Test of Independence between categorical clinical parameters

Var1	Var2	p	Test
GENDER	Ethnic	0.0001	Fisher's Exact Test
GENDER	first_drug	0.0370	Fisher's Exact Test
GENDER	Grandparents	0.0005	Fisher's Exact Test
GENDER	Smoking	0.0111	Fisher's Exact Test
GENDER	Motions_change	0.0128	Chi-Square Test
Ethnic	Grandparents	0.0005	Fisher's Exact Test
Ethnic	Motions_change	0.0181	Fisher's Exact Test
first_drug	Smoking	0.0060	Fisher's Exact Test
3rd_Drug	Smoking	0.0813	Chi-Square Test
Grandparents	Motions_change	0.0160	Fisher's Exact Test
Smoking	Motions_change	0.0182	Chi-Square Test

Table S7 : Pearson correlations continuous clinical parameters

Variable 1	Variable 2	R	*p*
Height	SAT	-0.3376	0.0003
SAT	Height	-0.3376	0.0003
BMI	Delta	0.3140	0.0007
Delta	BMI	0.3140	0.0007
AGE	Txn_AGE	0.3120	0.0010
Txn_AGE	AGE	0.3120	0.0010
Delta	SAT	0.2917	0.0019
SAT	Delta	0.2917	0.0019
Delta	Hip circumference	0.2746	0.0041
Hip circumference	Delta	0.2746	0.0041
Hip circumference	VAT	0.2146	0.0237
VAT	Hip circumference	0.2146	0.0237
Delta	Height	-0.2002	0.0324
Height	Delta	-0.2002	0.0324
Hip circumference	Txn_AGE	0.2054	0.0334
Txn_AGE	Hip circumference	0.2054	0.0334
BMI	Txn_AGE	0.1976	0.0376
Txn_AGE	BMI	0.1976	0.0376
AGE	SAT	-0.1943	0.0410
SAT	AGE	-0.1943	0.0410
Height	Txn_AGE	-0.1821	0.0506
Txn_AGE	Height	-0.1821	0.0506
AGE	BMI	-0.1702	0.0679
BMI	AGE	-0.1702	0.0679
Txn_AGE	VAT	0.1666	0.0806
VAT	Txn_AGE	0.1666	0.0806
AGE	Delta	-0.5346	0.0000
Delta	AGE	-0.5346	0.0000
Delta	Txn_AGE	0.6361	0.0000
Txn_AGE	Delta	0.6361	0.0000
Hip circumference	SAT	0.6827	0.0000
SAT	Hip circumference	0.6827	0.0000
BMI	SAT	0.7126	0.0000
SAT	BMI	0.7126	0.0000
BMI	Hip circumference	0.8383	0.0000
Hip circumference	BMI	0.8383	0.0000
BMI	VAT	0.4070	0.0000
VAT	BMI	0.4070	0.0000

Table S8: ANOVA Interactions and mediation analysis

Variable 1	Variable 2	p value
BMI	Txn_AGE	0.74525
GENDER	Txn_AGE	0.99975
Ethnic	Txn_AGE	0.67926
first_drug	Txn_AGE	0.08231
3rd_Drug	Txn_AGE	0.01458
SAT	Txn_AGE	0.52187
AGE	Txn_AGE	1.00000
Grandparents	Txn_AGE	0.71027
Height	Txn_AGE	0.77130
Hip circumference	Txn_AGE	0.92100
Smoking	Txn_AGE	0.93317
daily_use_television	Txn_AGE	0.92122
Motions_change	Txn_AGE	0.23694
Delta	Txn_AGE	0.99999
GENDER	BMI	0.99982
Ethnic	BMI	0.98780
first_drug	BMI	0.04828
3rd_Drug	BMI	0.08112
SAT	BMI	0.66642
AGE	BMI	0.35532
Grandparents	BMI	0.24197
Height	BMI	0.49673
Hip circumference	BMI	0.17152
Smoking	BMI	0.81049
daily_use_television	BMI	0.81036
Motions_change	BMI	0.28501
Delta	BMI	1.00000
Ethnic	GENDER	0.99993
first_drug	GENDER	0.99990
3rd_Drug	GENDER	1.00000
SAT	GENDER	0.99991
AGE	GENDER	0.99978
Grandparents	GENDER	0.99994
Height	GENDER	0.99999
Hip circumference	GENDER	0.99980
Smoking	GENDER	NA
daily_use_television	GENDER	0.99988
Motions_change	GENDER	0.99987
Delta	GENDER	0.99998
first_drug	Ethnic	0.25648
3rd_Drug	Ethnic	0.65774
SAT	Ethnic	0.11339

AGE	Ethnic	0.29960
Grandparents	Ethnic	0.03997
Height	Ethnic	0.36762
Hip circumference	Ethnic	0.93922
Smoking	Ethnic	0.35515
daily_use_television	Ethnic	0.04234
Motions_change	Ethnic	0.37113
Delta	Ethnic	0.99999
3rd_Drug	first_drug	0.12576
SAT	first_drug	0.05639
AGE	first_drug	0.71051
Grandparents	first_drug	0.21531
Height	first_drug	0.64091
Hip circumference	first_drug	0.13535
Smoking	first_drug	0.48155
daily_use_television	first_drug	0.26990
Motions_change	first_drug	0.03890
Delta	first_drug	0.99999
SAT	3rd_Drug	0.07466
AGE	3rd_Drug	0.11407
Grandparents	3rd_Drug	0.21941
Height	3rd_Drug	0.69380
Hip circumference	3rd_Drug	0.32549
Smoking	3rd_Drug	0.92951
daily_use_television	3rd_Drug	0.05784
Motions_change	3rd_Drug	0.82324
Delta	3rd_Drug	1.00000
AGE	SAT	0.35566
Grandparents	SAT	0.99775
Height	SAT	0.18240
Hip circumference	SAT	0.83489
Smoking	SAT	0.04624
daily_use_television	SAT	0.91423
Motions_change	SAT	0.55268
Delta	SAT	1.00000
Grandparents	AGE	0.34668
Height	AGE	0.59620
Hip circumference	AGE	0.18579
Smoking	AGE	0.37426
daily_use_television	AGE	0.46971
Motions_change	AGE	0.58336
Delta	AGE	1.00000
Height	Grandparents	0.16585
Hip circumference	Grandparents	0.20888
Smoking	Grandparents	0.01399

daily_use_television	Grandparents	0.24771
Motions_change	Grandparents	0.34255
Delta	Grandparents	0.99999
Hip circumference	Height	0.18186
Smoking	Height	0.14961
daily_use_television	Height	0.03129
Motions_change	Height	0.61986
Delta	Height	1.00000
Smoking	Hip circumference	0.93614
daily_use_television	Hip circumference	0.06222
Motions_change	Hip circumference	0.36221
Delta	Hip circumference	1.00000
daily_use_television	Smoking	0.56181
Motions_change	Smoking	0.65676
Delta	Smoking	1.00000
Motions_change	daily_use_television	0.32330
Delta	daily_use_television	1.00000
Delta	Motions_change	1.00000
